# Comparison of Ultrasound-Guided Core Needle Biopsy Under the Assistance of Hydrodissection With Fine Needle Aspiration in the Diagnosis of High-Risk Cervical Lymph Nodes: A Randomized Controlled Trial

**DOI:** 10.3389/fonc.2021.799956

**Published:** 2022-01-13

**Authors:** Dengke Teng, Chunhui Dong, Daju Sun, Zhuo Liu, Hui Wang

**Affiliations:** ^1^ Department of Ultrasound, China-Japan Union Hospital of Jilin University, Changchun, China; ^2^ Department of Pathology, China-Japan Union Hospital of Jilin University, Changchun, China; ^3^ Department of Gastrointestinal Colorectal and Anal Surgery, China-Japan Union Hospital of Jilin University, Changchun, China

**Keywords:** ultrasound, cervical lymph node, core needle biopsy, hydrodissection, fine needle aspiration

## Abstract

A randomized comparison of ultrasound (US)-guided core needle biopsy (CNB) under the assistance of hydrodissection with fine needle aspiration (FNA) was performed to evaluate the feasibility, safety and effectiveness for the diagnosis of high-risk cervical lymph nodes. Patients from December 2018 to May 2020 were randomly assigned to the CNB group and the FNA group at a ratio of 1:1. This study protocol was approved by the Ethics Committee of our hospital and registered in the Chinese Clinical Trial Registry (ChiCTR1800019370). The feasibility of CNB for high-risk cervical lymph nodes was evaluated by observing and recording the separation success rate (SSR) and technical success rate (TSR) of the CNB group. Safety was evaluated by comparing the incidence of major complications in the two groups. The diagnostic efficacy was evaluated by comparing the diagnostic accuracy, sensitivity, and specificity of the two groups. A total of 84 patients (84 lymph nodes) were randomized into the CNB (n = 42) and FNA (n = 42) groups. All patients in the CNB group achieved successful hydrodissection and biopsy. The SSR and TSR were both 100% in the CNB group. There were no major complications during or after the process in the two groups. Compared with the FNA group, the CNB group was significantly superior in terms of diagnostic accuracy and sensitivity (100% vs. 81.0%, *P* = 0.009; 100% vs. 79.2%, *P* = 0.035, respectively). The specificity of the two groups was 100%, and there was no significant difference. Compared with FNA, CNB under the assistance of hydrodissection is a feasible and safe method but is more effective for the diagnosis of high-risk cervical lymph nodes.

**Clinical Trial Registration:**

http://www.medresman.org, ChiCTR1800019370.

## Introduction

Cervical lymph nodes are a common metastatic site for a variety of malignant tumours (1–4). For suspicious cervical lymph nodes, timely and accurate qualitative diagnosis is of great usefulness for judging the prognosis and determining the choice of treatment (5). For qualitative diagnosis of cervical lymph nodes, a common method is ultrasound (US)-guided core needle biopsy (CNB). It is widely used due to its ease of processing and high diagnostic accuracy (6–9). However, when the cervical lymph nodes are adjacent to important structures such as large vessels and nerves, especially the relatively small lymph nodes, which are called “high-risk cervical lymph nodes”, CNB may cause serious complications such as blood vessel or nerve damage (10). At this time, fine needle aspiration (FNA), as a safer method, becomes an option. However, FNA has a low diagnostic accuracy rate and a high false-negative rate for some lymph node diseases (11–13), which fails to meet the needs of clinical diagnosis in some cases. Therefore, identifying a reliable diagnostic method that can take into account both safety and effectiveness is an urgent problem in the current diagnosis of high-risk cervical lymph nodes.

Hydrodissection, as a method to improve the safety of thermal ablation, was used to separate the target area and important structures by injection of normal saline. It has achieved outstanding results in the reduction of major complications of thermal ablation in previous studies (14–17). Based on this information, we inferred that hydrodissection may help to improve the safety of CNB in small high-risk cervical lymph nodes, thereby satisfying both safety and effectiveness. Moreover, to the best of our knowledge, few studies have reported the use of hydrodissection with cervical lymph node biopsy. Furthermore, there are few relevant randomized controlled trials comparing this technique with FNA. Therefore, we performed a randomized controlled trial to evaluate the feasibility, safety, and diagnostic effectiveness of CNB under the assistance of hydrodissection by comparing it with FNA in the diagnosis of high-risk cervical lymph nodes.

## Methods

### Trial Design

This was a prospective, randomized, controlled trial. According to a computer-generated randomization list, participants were randomized into the CNB (n = 42) and FNA (n = 42) groups. This study protocol was approved by the Ethics Committee of our hospital and registered in the Chinese Clinical Trial Registry (ChiCTR1800019370). Each patient fully understood the risks of puncture and signed an informed consent form before participating in the process.

The inclusion criteria were as follows (meeting all 3 of the following items): (1) US examination revealed cervical lymph nodes with suspicious malignant features (18, 19) such as hyperechoic, noncircumscribed margin, absence of hilum, gross necrosis, calcification, peripheral or mixed vascularity and shortest-to-longest axis ratio (S/L ratio) ≤0.5; (2) cervical lymph nodes were adjacent to important structures such as blood vessels and nerves; and (3) the maximum diameter of the cervical lymph nodes was ≤1.5 cm.

The exclusion criteria were as follows: (1) severe bleeding and coagulation disorders; (2) severe cardiorespiratory, nervous, liver, or kidney dysfunction; and (3) history of anaesthetic allergy.

#### Patients

From December 2018 to May 2020, a total of 88 consecutive patients who came to our hospital with suspicious high-risk cervical lymph nodes were enrolled, of whom 4 were lost to follow-up. Finally, 84 patients (84 lymph nodes) were included in our study. All patients were randomly assigned to the CNB group or the FNA group at a ratio of 1:1, with 42 cases in each group.

### Equipment

A colour Doppler US unit (Resona8, Mindray, China) with a 5–10 MHz linear array probe (L14-5WU, Mindray) was used to record images and guide the procedure. CNB was performed with an automatic biopsy needle gun (TSK, Japan). The gun had a needle length of 150 mm, a sampling length of 16 mm, and a sampling width of 1.6 mm. A 21-G needle (38 mm in length) with a 20-mL syringe (BD, USA) was used as the puncture needle for hydrodissection. A 22G fine aspiration cytology needle (Happo Co., Ltd, Japan) was used for FNA.

#### Preoperative Evaluation and Puncture Procedure

Before the process, all patients underwent US examination to assess the size, location, blood flow and relationship with the adjacent surrounding important structures, such as large blood vessels or nerves. Based on the above information, the process plan was designed. All CNB and FNA procedures were performed by two radiologists with more than 10 years of experience in intervention ultrasound. The patients’ necks were fully exposed, and the patients were placed in the supine or lateral position according to the location of the target node. The skin was sterilized and draped, and then local infiltration anaesthesia with 1% lidocaine was given.

In the CNB group, hydrodissection was performed with US guidance. A syringe needle was inserted between the target lymph node and the adjacent important structures. Then, the isolation fluid was injected to separate the soft tissue of the important structures from the lesion. The success of hydrodissection was defined as follows: after saline was injected between the target lymph node and nearby important structures, there was enough space that the biopsy gun would not damage the surrounding important structures when the biopsy gun was ejected. After successful hydrodissection, the volume of isolation fluid injected was recorded. Then, the biopsy gun penetrated the lesion for biopsy with the guidance of US. From each lymph node, 2 to 4 pieces of tissue was collected. Finally, the CNB process was finished after confirming that the sampling tissue was sufficient by the radiologists. Then, the tissue strips were placed in formalin solution.

In the FNA group, FNA was performed with US guidance. A 22G fine aspiration cytology needle was inserted into the target lymph node. Then, appropriate negative pressure suction was used to repeatedly lift and insert 20–30 times in different parts of the lymph node. After releasing the negative pressure, each sample was quickly mounted onto a glass slide for smearing. Four to six slides were used for each lymph node, and the slides were fixed in 95% ethanol. Finally, all samples were sent for cytological analysis.

After the process, both groups of patients pressed the puncture point by themselves for 30 minutes and were observed for 2 hours. Complications were recorded and classified as minor or major (20–23).

#### Pathological Evaluation

Each specimen was independently evaluated by two pathologists with more than 10 years of experience. When the evaluation results were not the same, they reviewed all the clinical data together and came to the final judgement. Pathological results were divided into positive malignant lymph nodes, negative malignant lymph nodes, and nondiagnostic results (insufficient samples or inability to rule out malignancy). Patients who were classified as negative malignant lymph nodes received a 6-month follow-up with intervals of 1, 3, and 6 months by US examination. During the follow-up, if the number of lymph nodes did not increase, the benign result of FNA/CNB was considered the final pathological result, and there was no need for another CNB. In contrast, another CNB was needed to confirm the final pathological result when the following conditions occurred: (1) patients with negative malignant lymph nodes with enlargement during the follow-up, (2) patients with negative malignant lymph nodes but unclear pathological types, (3) patients with nondiagnostic results, and (4) patients who had a known primary tumour, but the pathological type of the positive malignant lymph node was inconsistent with the primary tumour.

### Outcomes

The feasibility of CNB for high-risk cervical lymph nodes was evaluated by observing and recording the separation success rate (SSR) and technical success rate (TSR) of the CNB group. SSR was defined as the ratio of the number of patients with successful hydrodissection to the total number of patients in the CNB group. Technical success was defined as the biopsy gun being correctly inserted into the target position, with CNB being completed according to the preset plan. TSR was defined as the ratio of the number of patients with successful technology to the total number of patients in the CNB group.

Safety was evaluated by comparing the incidences of major complications in the two groups.

The diagnostic efficacy was evaluated by comparing the diagnostic accuracy, sensitivity, and specificity of the two groups.

#### Sample Size and Statistical Analysis

Based on previous studies (24, 25), the sensitivity of FNA in diagnosing malignant cervical lymph nodes was 66.7%, and the sensitivity of CNB in diagnosing malignant cervical lymph nodes was 96.8%. When using a two-sided 5% type I error and 95% statistical power, 74 patients were required. Assuming a loss to follow-up rate of 10%, we set the final sample size to at least 41 patients in each group.

SPSS26.0 was used to analyse the data. The sex, location, diagnostic accuracy, sensitivity, specificity, and complication rate of the two groups were compared with the chi-square test. Measurement data such as age and the maximum diameter of cervical lymph nodes were described by the mean ± SD, and the differences between the two groups were compared with *t* tests. A *P* value < 0.05 was considered statistically significant.

## Result

A total of 84 patients (84 lymph nodes) were included in the study. In the CNB group, there were 42 patients, including 22 males and 20 females. The age of the patients was 62 ± 12 years (range 26–78 years), and the maximum diameter of the lymph nodes was 1.24 ± 0.26 cm (range 0.50–1.50 cm). In the FNA group, there were a total of 42 patients, including 22 males and 20 females. The age of the patients was 56 ± 16 years (range, 16–79 years), and the maximum diameter of the lymph nodes was 1.23 ± 0.2 cm (range, 0.53–1.50 cm). There was no statistically significant difference in basic information (**Table 1**) between the two groups of patients (*P* > 0.05).

**Table 1:** Basic information of the patients in the FNA and CNB groups.

	FNA	CNB	*P* value
Total patients	42	42	–
Sex (Male/Female)	22/20	22/20	0.929
Age (years)	56 ± 16 (16–79)	62 ± 12 (26–78)	0.074
Lymph node site (left/right)	23/19	17/25	0.168
Maximum diameter (cm)	1.23 ± 0.26 (0.53–1.50)	1.24 ± 0.26 (0.50–1.50)	0.751

CNB, core needle biopsy; FNA, fine needle aspiration.

### The Feasibility in the CNB Group

All patients in the CNB group achieved successful hydrodissection between the target lymph node and adjacent important structures, such as large vessels and nerves. In addition, the SSR was 100% (42/42). The average volume of the injected saline was 17 ± 5.8 ml (range, 5–35 ml). All high-risk cervical lymph nodes successfully underwent CNB. In addition, the TSR was 100%. The length of the tissue strip was 0.30~1.30 cm, and the number of sampled tissue strips was 2~4.

#### Complications

In the CNB group, two patients had slight swelling at the puncture site during the injection of isolation fluid, and the symptoms disappeared on their own after 30 minutes. There were no major complications during or after the process in either group.

##### Diagnostic Effectiveness

###### 3.2.1 CNB Group

Of the 42 patients undergoing CNB under the assistance of hydrodissection, the CNB results showed that 29 patients (69.0%) had positive malignant lymph nodes and 13 patients (31.0%) had negative malignant lymph nodes. All patients received a clear diagnosis and pathological type in the biopsy, and the pathological type was consistent with the pathological type of the primary tumour. At the same time, among the 13 patients with negative malignant lymph nodes, there were no patients with enlarged lymph nodes during the follow-up period. Therefore, no cases required a second biopsy in the CNB group (**Table 2**). Based on these results, we defined the CNB results as the final pathological diagnosis.

**Table 2:** Pathological diagnosis in the FNA and CNB groups.

	FNA (case)		CNB (case)	
	First	Final	First	Final
Malignant positive	19 (45.2%)	24 (57.1%)	29 (69.0%)	29 (69.0%)
Metastatic	19	21	26	26
Lymphoma	0	3	3	3
Malignant negative	21 (47.6%)	18 (42.9%)	13 (31.0%)	13 (31.0%)
Benign lymphoid hyperplasia	5	5	9	9
Tuberculosis	7	11	4	4
Lymphadenitis	0	1	0	0
Schwannoma	0	1	0	0
Unclear pathology	9	0	0	0
Insufficient sample	2 (4.8%)	0	0	0

CNB, core needle biopsy; FNA, fine needle aspiration.

###### FNA Group

Of the 42 patients undergoing FNA, 19 patients (45.2%) had positive malignant lymph nodes, 21 patients (50%) had negative malignant lymph nodes, and 2 patients (4.8%) had nondiagnostic results (insufficient samples). According to the FNA results, 12 cases required a second biopsy, and 9 cases required follow-up (**Table 3**). Among the 9 patients requiring follow-up, no cases required a second biopsy due to an increase in volume during the follow-up period. Based on the results of the second biopsy and follow-up, we concluded that the final pathological results of the FNA group were as follows: 24 cases were positive malignant lymph nodes, and 18 cases were negative malignant lymph nodes (**Tables 2** and **3**).

**Table 3:** Pathological diagnosis of patients undergoing a second biopsy in the FNA group.

Pathological diagnosis		
First (case)		Final (case)
Squamous cell carcinoma (1)		Adenoid cystic carcinoma (1)
Insufficient sample (2)		Schwannomas (1)
		Tuberculosis (1)
Negative malignant lymph nodes with no specific pathological type (9)		Squamous cell carcinoma (2)*
		Lymphoma (3)*
		Tuberculosis (3)
		Necrotizing lymphadenitis (1)

Five cases of false negatives were found in the FNA group (*).

CNB, core needle biopsy; FNA, fine needle aspiration.

##### General Results

**Figure 1:**
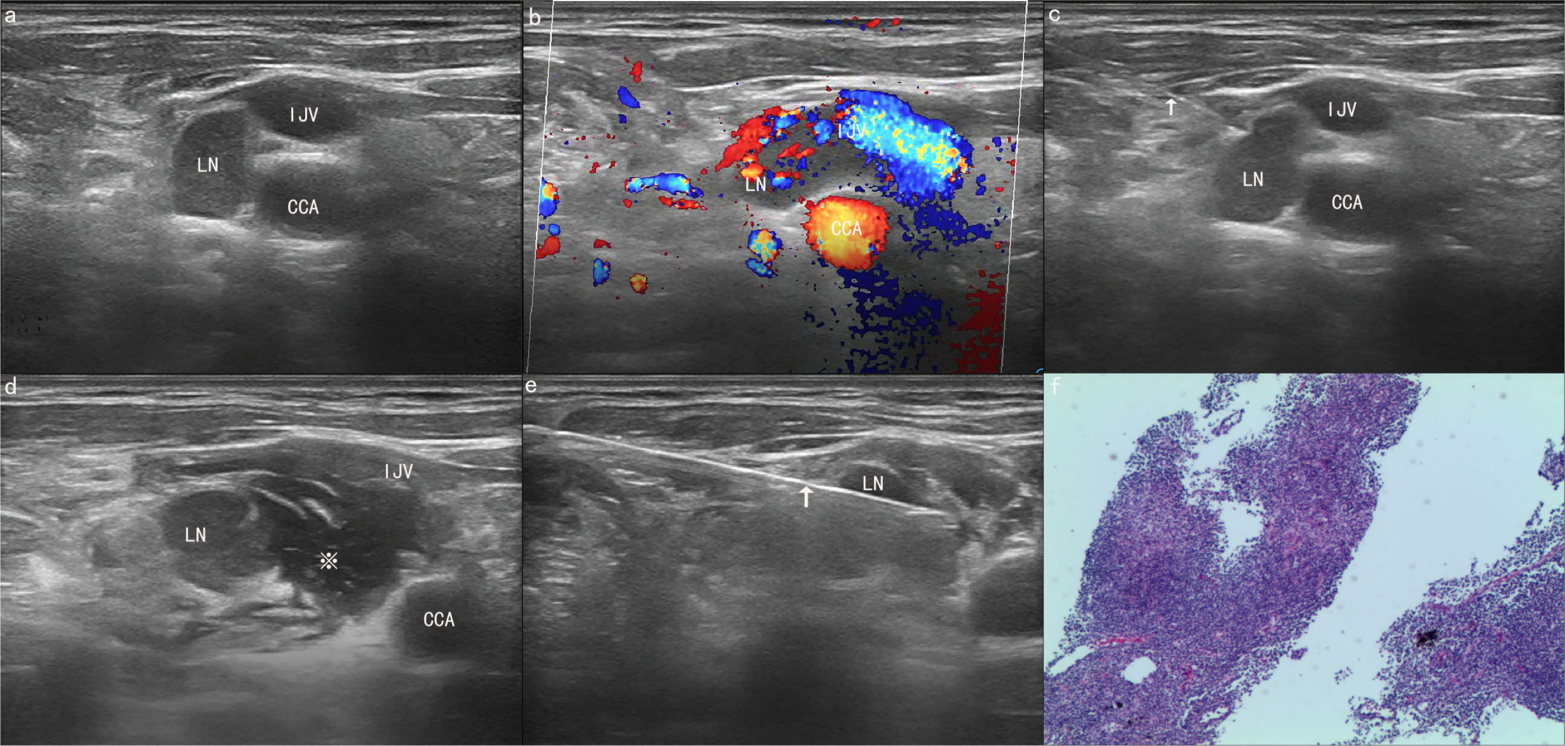
A 66-year-old man was found to have a suspicious and enlarged lymph node (LN) in the right neck during an ultrasound (US) examination. **(A)** The ultrasound image showed that a lymph node with a maximum diameter of 1.46 cm was found in area IV of the right neck, adjacent to the common carotid artery (CCA) and internal jugular vein (IJV). **(B)** The colour Doppler flow image (CDFI) shows the blood flow signal of the lesion and blood vessel. **(C)** A 21G needle (arrow) was used to puncture the edge of the lymph node for hydrodissection. **(D)** After hydrodissection was completed successfully, the lymph node and CCA were filled with a large amount of normal saline (asterisk). **(E)** Core needle biopsy (CNB) was performed safely with an 18G needle (arrow). **(F)** The final pathological result proved to be lymphadenitis. The tissue was stained with haematoxylin and eosin (×40).

Based on these results, the diagnostic efficiency of the CNB group was as follows: the diagnostic accuracy, sensitivity and specificity were 100%, 100%, and 100%, respectively. The diagnostic efficiency of the FNA group was as follows: the diagnostic accuracy, sensitivity and specificity were 81.0%, 79.2%, and 100%, respectively. Generally, compared with the FNA group, the diagnostic effectiveness of the CNB group was superior to that of the FNA group in terms of diagnostic accuracy and sensitivity, which were significantly different (100% vs. 81.0%, *P* = 0.009; 100% vs. 79.2%, *P* = 0.035, respectively) (**Table 4**). **Figure 1** show the US presentations during Ultrasound-Guided Core Needle Biopsy Under the Assistance of Hydrodissection with patient of lymphadenitis.

**Table 4:** Diagnostic efficacy for lymph nodes in the two groups.

	FNA group	CNB group	*P* value
Diagnostic accuracy	81.0% (34/42)	100% (42/42)	0.009
Sensitivity	79.2% (19/24)	100% (29/29)	0.035
Specificity	100% (18/18)	100% (13/13)	–

CNB, core needle biopsy; FNA, fine needle aspiration.

## Discussion

This randomized controlled study showed that CNB under the assistance of hydrodissection was feasible and safe for cervical lymph node biopsy in high-risk areas. It had a better diagnostic performance than FNA. Cervical malignant lymph nodes were often found in the presence of primary malignancy, mid-lower neck localization as Level 3-6, and markedly hypoechoic lymph nodes with loss of echo-genic hilum (26). The involvement of the central neck should be a major indication of lymph node biopsy and pathological diagnosis regardless of the imaging findings. In the lateral compartment, hypoechogenicity with loss of hilum, microcalcifications, cystic parts and an index value ≥ 0.51 are indications of a lymph node biopsy to rule out malignancy. (27). However, how to perform puncture biopsy safely and effectively for high-risk cervical lymph nodes is an urgent problem to be solved (27).

The results demonstrated that CNB under the assistance of hydrodissection was feasible for high-risk cervical lymph nodes. In our study, the results showed that in the CNB group, all patients successfully underwent effective hydrodissection and obtained a sufficiently safe puncture distance. The SSR was 100%, which was close to Cheng’s research results (28). The possible reasons were as follows: first, we injected enough saline during the hydrodissection because the neck tissue was loose and the injected saline was easily absorbed and diffused. Sufficient saline injection ensured sufficient separation and protection. The average volume of saline injected in this study was 17 ± 5.8 ml (range 5–35 ml), which was similar to Cheng’s results (28). Second, when injecting the isolation fluid, it should be injected along the edge of the target lymph node under real-time US guidance, which could improve the efficiency of separation. Finally, for patients who have undergone other treatments, such as surgery and radiotherapy, that can cause adhesions in the cervical tissues, it was difficult to use a 10- or 20-ml syringe to separate the cervical lymph nodes from the surrounding tissues. A smaller syringe needle, such as 2 ml or 5 ml, was used to inject the isolation fluid, which could help to improve the SSR. In addition, enough specimen for pathological diagnosis after hydrodissection was obtained from all of the lymph nodes in the CNB group. An automatic biopsy gun is helpful for some small and hard lymph nodes, which tend to move during the puncture process. This benefit is due to the large ejection force of the automatic biopsy gun, which can complete the sampling process before the lymph node moves.

This study showed that there was no significant difference in the safety of CNB under the assistance of hydrodissection compared with FNA. The successful application of hydrodissection provides sufficient operating space for CNB, making difficult cases easier and even making impossible cases possible most of the time. Both groups of patients had no major complications, such as large blood vessel or nerve damage, during and after the process. Although 2 patients in the CNB group experienced swelling during the hydrodissection, these cases resolved completely on their own within 30 minutes.

The study showed that CNB was better than FNA with respect to the diagnostic efficacy of high-risk cervical lymph nodes. The results showed that the diagnostic accuracy and sensitivity of the CNB group were as high as 100%, which was significantly higher than that of the FNA group (83.3% and 79.2%, respectively). This result was similar to Xu’s result (29). The possible reasons were as follows. First, CNB was more effective than FNA in the diagnosis of malignant diseases. All malignant lymph nodes in the CNB group were accurately diagnosed. However, the FNA group had low sensitivity for lymphoma. In our study, all 3 lymphomas in the FNA group were not correctly diagnosed, which was similar to the results of Lioe’s analysis (30). Second, CNB is also better than FNA in diagnosing benign lymph nodes. All benign lymph nodes in the CNB group were accurately diagnosed. In contrast, among the 11 cases of tuberculosis in the FNA group, 4 cases were not accurately diagnosed. Finally, CNB was superior to FNA in the diagnosis of the pathological typing of cervical lymph nodes. All lymph nodes in the CNB group were correctly pathologically typed. In contrast, there were 9 cases of negative malignant lymph nodes in the FNA group that had not been clearly pathologically typed. Of these 9 cases, 5 cases were diagnosed as positive for malignant lymph nodes at the second biopsy.

This study still has some limitations. First, our study was a single-centre study with a small sample size. Second, the final pathological results were based on CNB and follow-up, and there was no excisional biopsy, which represents the gold standard. Third, the follow-up time was only 6 months, and it was not enough to make an accurate and objective evaluation for certain slow-growing malignant lymph nodes.

## Conclusion

Compared with FNA, US-guided CNB under the assistance of hydrodissection is a feasible, safe, and more effective method for the diagnosis of high-risk cervical lymph nodes.

## Data Availability Statement

The original contributions presented in the study are included in the article/supplementary material. Further inquiries can be directed to the corresponding authors.

## Ethics Statement

This study protocol was approved by the Ethics Committee of our hospital and registered in the Chinese Clinical Trial Registry (ChiCTR1800019370). Written informed consent to participate in this study was provided by the participants’ legal guardian/next of kin.

## Author Contributions

DT and DS contributed to conception and design of the study. CD organized the database, performed the statistical analysis and wrote the first draft of the manuscript. All authors contributed to manuscript revision, read, and approved the submitted version.

## Funding

The study was supported by the China Postdoctoral Science Foundation (2019M661219) and the Finance Department of Jilin Province (2020SCZ08).

## Conflict of Interest

The authors declare that the research was conducted in the absence of any commercial or financial relationships that could be constructed as a potential conflict of interest.

## Publisher’s Note

All claims expressed in this article are solely those of the authors and do not necessarily represent those of their affiliated organizations, or those of the publisher, the editors and the reviewers. Any product that may be evaluated in this article, or claim that may be made by its manufacturer, is not guaranteed or endorsed by the publisher.
